# Supplementary material for: Revealing the biexciton and trion-exciton complexes in BN encapsulated WSe2
Source: Nat Commun. 2018 Sep 13;9:3719. doi: 10.1038/s41467-018-05863-5 (PMC6137082; doi:10.1038/s41467-018-05863-5)
Supplement: Supplementary file 1 — Supplementary Information [file 41467_2018_5863_MOESM1_ESM.pdf]

Supplementary Information

Revealing the Biexciton and Trion-exciton Complexes in BN Encapsulated WSe_2

Zhipeng Li[1,2,#], Tianmeng Wang[1,#], Zhengguang Lu[3,4], Chenhao Jin[5], Yanwen Chen[1], Yuze Meng[1,6], Zhen Lian[1], Takashi Taniguchi[7], Kenji Watanabe[7], Shengbai Zhang[8], Dmitry Smirnov[3], Su-Fei Shi[1,9*]

1. Department of Chemical and Biological Engineering, Rensselaer Polytechnic Institute, Troy, NY 12180
2. School of Chemistry and Chemical Engineering, Shanghai Jiao Tong University, Shanghai, 200240, China
3. National High Magnetic Field Lab, Tallahassee, FL, 32310
4. Department of Physics, Florida State University, Tallahassee, Florida 32306, USA
5. Physics Department, University of California, Berkeley, CA 94720
6. College of Physics, Nanjing University, Nanjing, 210093, P. R. China
7. National Institute for Materials Science, 1-1 Namiki, Tsukuba 305-0044, Japan.
8. Department of Physics, Applied Physics, and Astronomy, Rensselaer Polytechnic Institute, Troy, NY 12180
9. Department of Electrical, Computer & Systems Engineering, Rensselaer Polytechnic Institute, Troy, NY 12180

[#] These authors contributed equally to this work

[*] Corresponding author: shis2@rpi.edu

Supplementary Note 1. Sample preparation

The BN encapsulated single layer WSe_2 device was fabricated by the dry transfer technique: PPC (poly propylene carbonate) pickup method[1]. First, the single layer WSe_2, few-layer graphene and few-layer BN were exfoliated onto Si substrate with 300 nm thick SiO_2 and inspected by the optical microscope. A microscope slide with ~1 μm thick PPC on the transparent elastomer stamp (PDMS, poly dimethyl siloxane) was inverted and attached to the micromanipulator of the transfer stage. We used the manipulator to align and pick up the few-layer BN flake. This process was repeated to pick up the single layer WSe_2, few-layer graphene and another few-layer BN in sequence. The prepared stack was placed onto a pair of pre-patterned Au electrodes and removed the PPC by heating up to 90 °C. The PPC residue was removed by chloroform to leave the BN/WSe_2/Graphene/BN stack on the substrate. Finally, we added another few-layer graphene onto

the top BN as a top gate electrode, using the top BN as the gate dielectrics.

It is worth to note that h-BN has rich spectroscopic features in the visible regime due to defect states. However, the related PL is much weaker than that of the monolayer WSe_2. Our high-quality WSe_2 device also enables the onset of the biexciton behavior at low excitation power, under which our control measurements of the bare BN flakes did not show any observable PL, confirming that the measured PL from the BN encapsulated monolayer WSe_2 is from WSe_2.

We have also extended our fabrication method to investigate the BN encapsulated monolayer WS_2, and the low-temperature PL as a function of the excitation power (CW 532 nm laser) (Supplementary Figure 1). Different from WSe_2, the WS_2 remains n-doped and cannot be tuned into intrinsic or p-doped through the gate voltage. As a result, the biexciton PL cannot be observed. However, the exciton (X_0), splitted n-trions (X_1^- and X_2^-) and charged biexciton (XX^-) can be clearly resolved. The corresponding PL peaks are intrinsic to the monolayer WS_2, and are evidently different from the PL from the BN encapsulated WSe_2 in Fig. 1C of the main text. The apparently different energy of the XX^- in BN encapsulated WS_2, compared with that from the WSe_2, confirming that the emerging PL is indeed from center TMDC monolayer encapsulated by the BN, not from the contact with the BN.

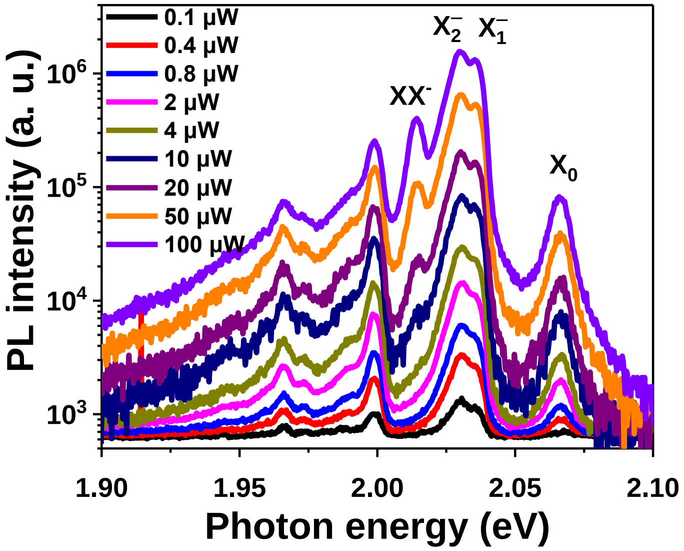

Supplementary Figure 1. PL spectra of BN encapsulated WS_2 as a function of the excitation (CW 532 nm laser) power.

Supplementary Note 2. Magneto-optical measurement setup

The magneto-PL setup is shown in Figure S1. The incident laser was focused by a 32X

objective (NA: ~0.6) to a spot size of ~2 μm. For the lifetime measurement, a 40 MHz supercontinuum laser covering the 410~2400 nm was used as the excitation source.

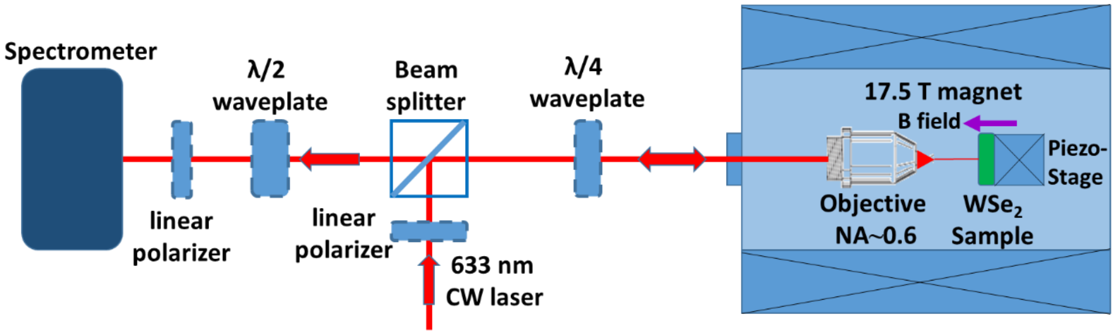

Supplementary Figure 2. Schematic of the magneto-optical setup for the photoluminescence measurement

Supplementary Note 3. $\boldsymbol{g}$**-factor calculation**

A general formula for the g-factor of each band can be expressed in terms of three parts based on a tight binding model[2–4]: orbital contribution g_O, valley contribution g_V, and spin contribution g_S.

The Zeeman energy split is denoted by:

$$\Delta = E^K - E^{K'} = (\tau_z g_V + \tau_z g_O + \sigma_z g_S)\,\mu_B B \quad (1),$$

where $\sigma_z = +1\ (-1)$ is for spin-up (spin-down) configuration, τ_z represents the valley degree of freedom, with $\tau_z = +1\ (-1)$ for K (K') valley.

With a simplified model[5,6] in which only d-orbital components are considered in both the valence band and conduction band of monolayer WSe_2, the orbital, valley and spin contribution to the g-factor is 4, $2\,(m_0/m_h)$, 2 for the valence band and 0, $2\,(m_0/m_e)$, 2 for the conduction band, respectively, where m_0 is the mass for the free electron, m_h is the effective mass of the hole and m_e is the effective mass of the electron. As a result, the bright exciton should have the total g-factor:$g^t = -4 + 2\,(m_0/m_e - m_0/m_h)$, which is -4 when $m_e = m_h$. The spectral g-factor, as discussed in the main text, should be the same, with $g^t = -4$. The calculated g-factors and configurations for exciton, dark exciton, trion-exciton complexes and biexciton are shown in Supplementary Table 1 and Supplementary Figure 3.

Supplementary Table 1. The calculated *g*-factors for the exciton, dark exciton, trion-exciton complex and biexciton.

Peaks	Total *g*-factor	Spectral *g*-factor
**Bright exciton (X_0)**	-4.0	-4.0
**Dark exciton (D)**	-8.0	-8.0
**Trion-exciton complexes (XX^-)**	6.0	-4.0
**Biexciton (XX) Fig. 3(d)**	4.0	-4.0
**Biexciton (XX) Fig. 3(e)**	0.0	-4.0

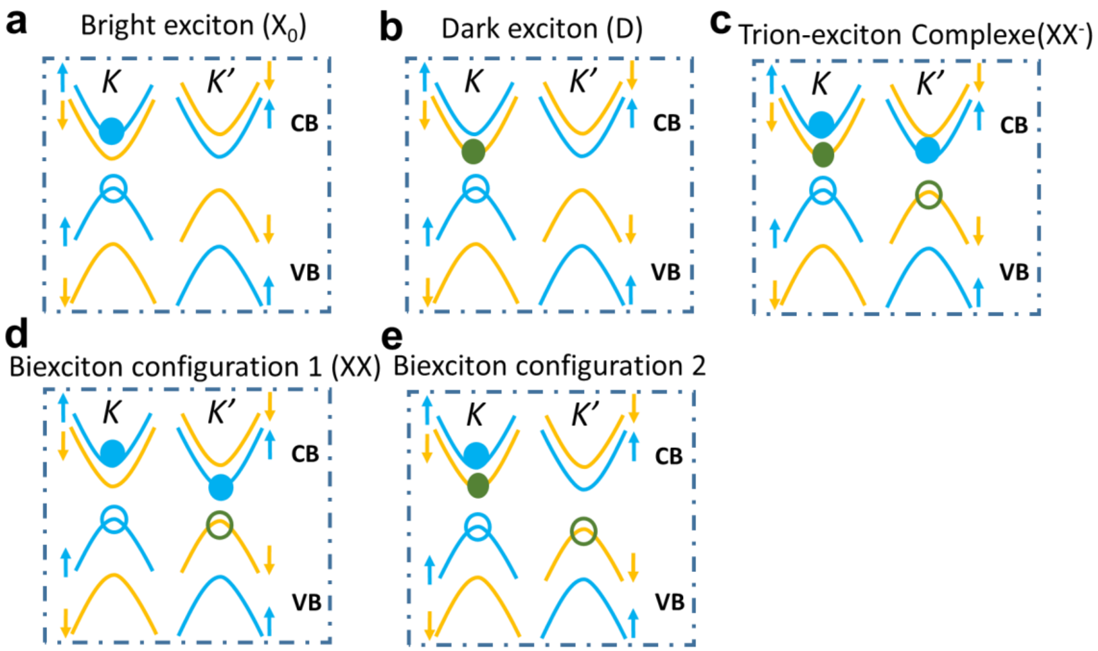

Supplementary Figure 3. The configuration for the exciton, dark exciton, trion, biexciton and trion-exciton. (a) The congfiguration for the bright exciton (X_0). (b) The congfiguration for the dark exciton (D). (c) The congfiguration for the (negative) trion-exciton complex (XX^-). (d) The congfiguration for the biexciton configuration 1 (XX) as shown in the main text. (e) The congfiguration for an alternative biexciton configuration (XX) (configuration 2). The configuration 2, as shown in table S1, has a total *g*-factor: $g^t = 0$, which cannot explained the intensity ratio

observed in the magneto-PL spectra. The configuration 2 is hence ruled out as a possible configuration for the biexciton.

Supplementary Note 4. Spectral g-factor: g^S

Alternatively, the spectral g-factor can also be obtained through the fitting of Zeeman shift ($\Delta^{\sigma^+\sigma^+} = \frac{1}{2}\mu_B g B$, $\Delta^{\sigma^-\sigma^-} = -\frac{1}{2}\mu_B g B$) as a function of B field for either $\sigma^+\sigma^+$ (Supplementary Figure 4a) or $\sigma^-\sigma^-$ (Supplementary Figure 4b) configuration. The fitting results from these two configurations, show in Supplementary Figure 4c and Supplementary Figure 4d, agrees with each other. Bot results are also in good agreement with what is shown in the main text, which is obtained using the Zeeman splitting between the $\sigma^+\sigma^+$ and $\sigma^-\sigma^-$ configuration (Fig. 4c).

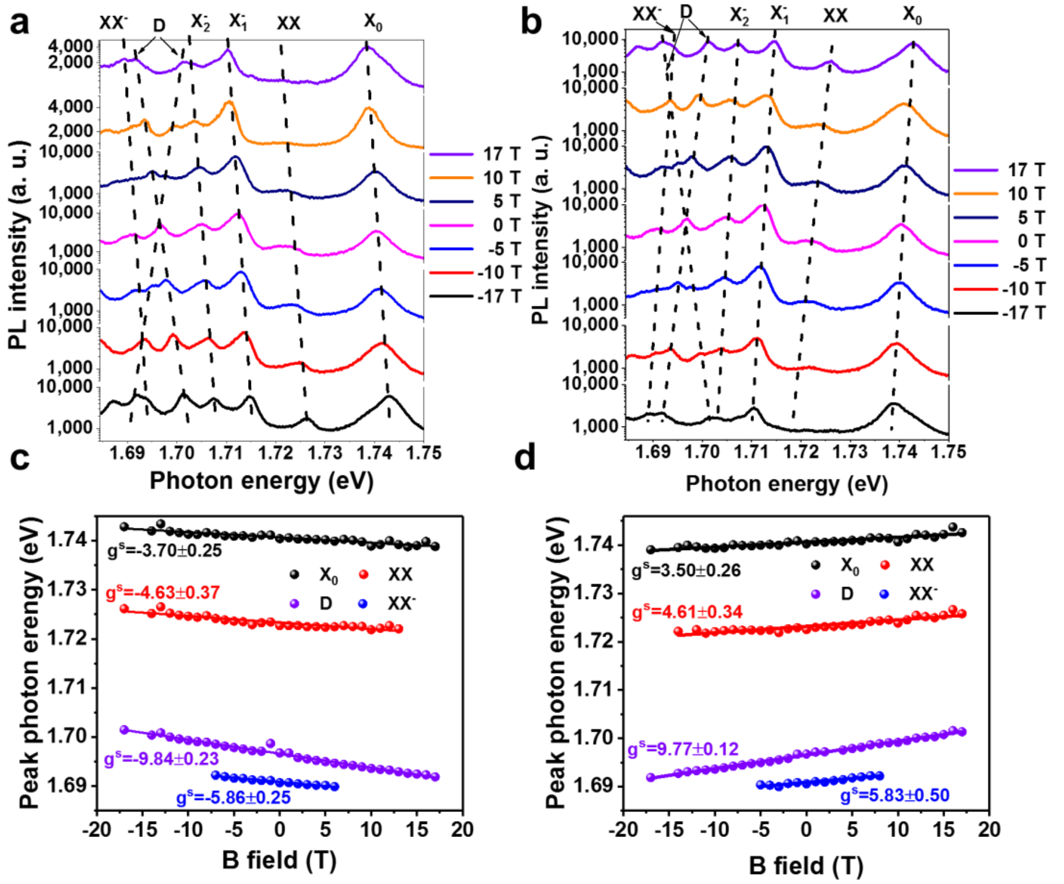

Supplementary Figure 4. Spectral g-factor calculations based on the PL Zeeman shift (a) PL spectra ($\sigma^+\sigma^+$) at 17 T, 10 T, 5 T, 0 T, -5 T, -10 T and -17 T, respectively. (b) PL spectra ($\sigma^-\sigma^-$) at 17 T, 10 T, 5 T, 0 T, -5 T, -10 T and -17 T, respectively. (c) g-factors for different peaks calculated from the Zeeman shift in (a). (d) g-factors for different peaks calculated from the Zeeman shift in

(b).

Supplementary Note 5. Time-resolved PL for the exciton, biexciton, trions and dark exciton.

For the WSe_2 device shown in Fig. 2 in the main text, we have measure the time-resolved PL in home built confocal microscope setup via time-correlated single photon counting (TCSPC) technique. The TRPL spectra (Supplementary Figure 5a) was measured using a 633 nm excitation, filtered (~ 6 nm bandpass filter) from a supercontinuum laser source (Fianium) with the repetition rate of 40 MHz. The excitation power is ~ 40 µW, with a focus spot of ~ 2 µm. The lifetime of the biexciton is comparable to that of the trions, confirming that the PL from XX is not from defects. The lifetime from dark exciton significantly longer than other peak, ~ 4 ns. We have used the response of the laser as a kernel and obtained the lifetimes of the bright exciton to be ~ 15 ps.

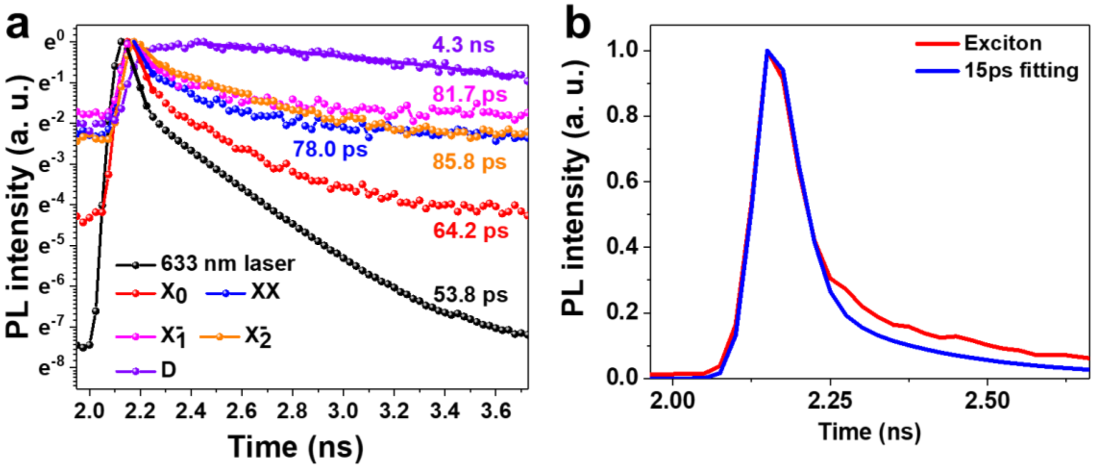

Supplementary Figure 5 (a) Time-resolved PL traces taken at 12 K for X_0, XX, X_1^-, X_2^- and dark exciton species, along with the instrument response function (excitation laser at 633 nm). A single exponential function $I = Ae^{-t/\tau_0}$ was used to fit the fast component of the decay. The results of τ_0 are shown in (a). (b) The extracted lifetime of the bright exciton using the response of the laser as a kernel.

Supplementary Note 6. Gate-voltage dependent PL spectra of the first WSe_2 device.

The gate voltage dependence of the PL spectra of the first WSe_2 device, the data of which is shown in Fig. 1, is plotted in Supplementary Figure 6. Supplementary Figure 6a shows similar behavior as the Fig. 2b in the main text, confirming the existent of the biexciton in the charge neutral WSe_2 and the (negative) trion-exciton complex in the lightly-doped WSe_2. The PL traces of the line cuts in Fig. S6a are shown in Supplementary Figure 6b. The Integrated PL intensity of X_0, D, XX, XX^-, and X_1^- as a function of the top gate voltage extracted from Fig. 2a is shown

in Supplementary Figure 6c.

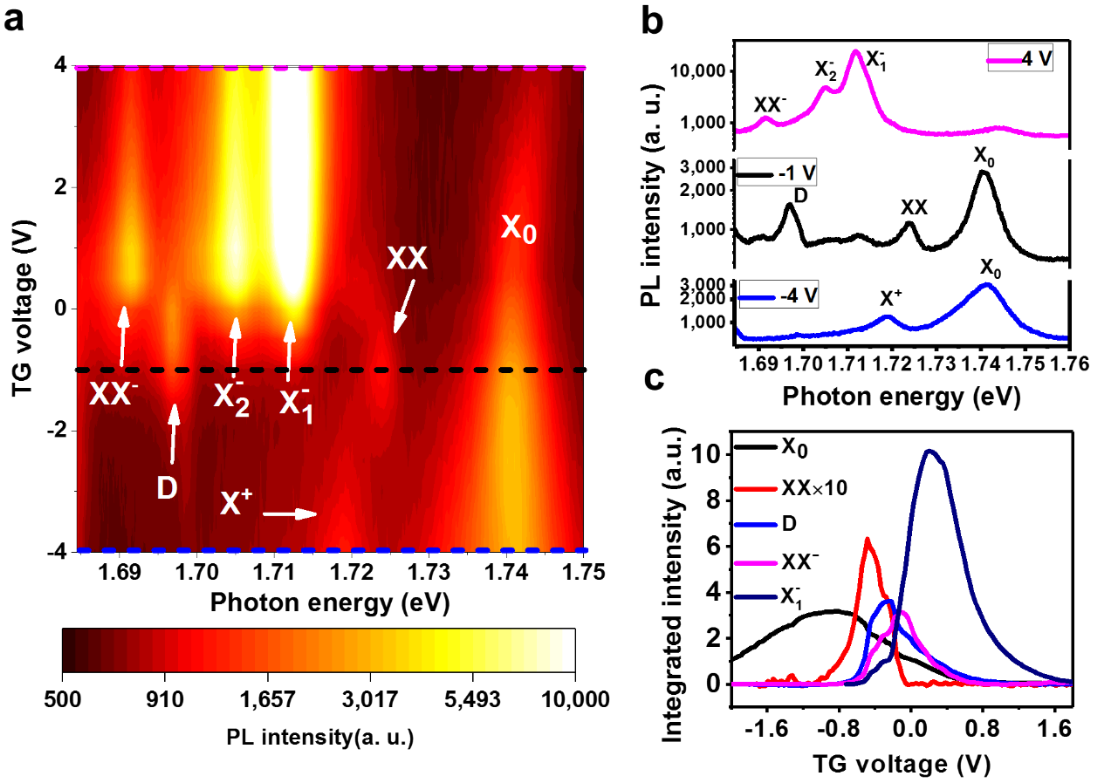

Supplementary Figure 6. PL spectra of the first monolayer WSe_2 device as a function of the top gate voltage (a) Color plot of the PL spectra as a function of the top gate voltage. The color represents the PL intensity. (b) PL spectra at specific top gate voltages. (c) Integrated PL intensity for X_0, D, XX, XX^-, and X_1^- as a function of the top gate voltage extracted from Fig. 2(a).

Supplementary Note 7. The Lorentzian fitting for the valley polarization calculation.

To calculate the valley polarization as shown in the Fig. 3a in the main text, we have used *Lorentzian* fitting to fit the PL spectra taken at $\sigma^+\sigma^+$ and $\sigma^+\sigma^-$ configurations (Fig. 3a of the main text).

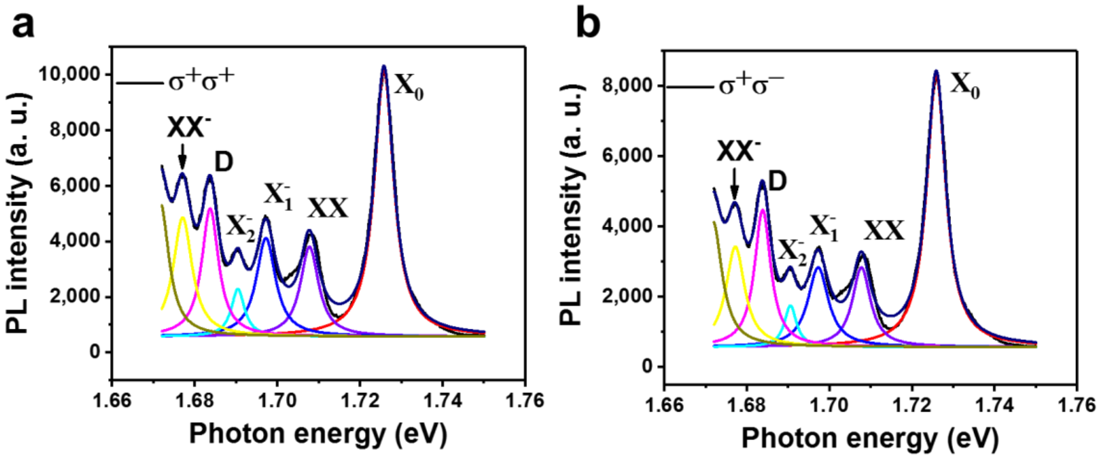

Supplementary Figure 7. The Lorentzian fitting of the PL spectra with a circularly polarized (σ^+) excitation and the same (σ^+) (a) or opposite (σ^-) (b) helicity detection. The different colors represent different the fitting for different peaks. The valley degree calculation is based on the integrated peak intensities of the fitting results.

Supplementary References

1. Wang, L. *et al.* One-Dimensional Electrical Contact to a Two-Dimensional Material. *Science (80-.).* **342,** 614–617 (2013).
2. Macneill, D. *et al.* Breaking of valley degeneracy by magnetic field in monolayer MoSe2. *Phys. Rev. Lett.* **114,** 1–5 (2015).
3. Srivastava, A. *et al.* Valley Zeeman effect in elementary optical excitations of monolayer WSe2. *Nat Phys* **11,** 141–147 (2015).
4. Aivazian, G. *et al.* Magnetic control of valley pseudospin in monolayer WSe2. *Nat Phys* **11,** 148–152 (2015).
5. Nagler, P. *et al.* Giant magnetic splitting inducing near-unity valley polarization in van der Waals heterostructures. *Nat. Commun.* **8,** 1–6 (2017).
6. Andreas V. Stier, Kathleen M. McCreary, Berend T. Jonker, J. K. & S. A. C. Exciton diamagnetic shifts and valley Zeeman effects in monolayer WS2 and MoS2 to 65Tesla. *Nat. Commun.* **33,** 416–420 (2016).
